# Using high throughput microtissue culture to study the difference in prostate cancer cell behavior and drug response in 2D and 3D co-cultures

**DOI:** 10.1186/s12885-018-4473-8

**Published:** 2018-05-24

**Authors:** Eman Mosaad, Karen Chambers, Kathryn Futrega, Judith Clements, Michael Robert Doran

**Affiliations:** 1 Stem Cell Therapies Laboratory, Queensland University of Technology (QUT), Translational Research Institute (TRI), 37 Kent Street, Brisbane, QLD Australia; 2 Australian Prostate Cancer Research Centre – Queensland (APCRC-Q), Translational Research Institute (TRI), Brisbane, Australia; 3 Biochemistry division, Chemistry Department, Faculty of Science, Damietta University, Damietta, Egypt; 4 Quadram Institute Bioscience, Norwich Research Park, Norwich, UK; 5 Mater Research Institute – University of Queensland, Translational Research Institute (TRI), Brisbane, Australia; 6 Australian National Centre for the Public Awareness of Science, Australian National University, Canberra, Australia

**Keywords:** Prostate cancer, Bone marrow stromal cells, Co-culture, Microwell-mesh platform, 3D culture

## Abstract

**Background:**

There is increasing appreciation that non-cancer cells within the tumour microenvironment influence cancer progression and anti-cancer drug efficacy. For metastatic prostate cancer (PCa), the bone marrow microenvironment influences metastasis, drug response, and possibly drug resistance.

**Methods:**

Using a novel microwell platform, the *Microwell-mesh*, we manufactured hundreds of 3D co-culture microtissues formed from PCa cells and bone marrow stromal cells. We used luciferase-expressing C42B PCa cells to enable quantification of the number of PCa cells in complex microtissue co-cultures. This strategy enabled us to quantify specific PCa cell growth and death in response to drug treatment, in different co-culture conditions. In parallel, we used Transwell migration assays to characterize PCa cell migration towards different 2D and 3D stromal cell populations.

**Results:**

Our results reveal that PCa cell migration varied depending on the relative aggressiveness of the PCa cell lines, the stromal cell composition, and stromal cell 2D or 3D geometry. We found that C42B cell sensitivity to Docetaxel varied depending on culture geometry, and the presence or absence of different stromal cell populations. By contrast, the C42B cell response to Abiraterone Acetate was dependent on geometry, but not on the presence or absence of stromal cells.

**Conclusion:**

In summary, stromal cell composition and geometry influences PCa cell migration, growth and drug response. The Microwell-mesh and microtissues are powerful tools to study these complex 3D interactions.

**Electronic supplementary material:**

The online version of this article (10.1186/s12885-018-4473-8) contains supplementary material, which is available to authorized users.

## Background

Despite significant improvements in the survival of prostate cancer (PCa) patients with localized disease, survival drops significantly if the cancer has metastasized to a distal site [1]. Approximately 90% of metastatic prostate cancer patients suffer bone metastasis [2, 3], making modeling of PCa cell behavior within the bone tissue microenvironment especially relevant.

Within the bone, there is evidence that the first site of metastasis is the hematopoietic stem cell (HSC) niche [1]. Key HSC niche microenvironmental cell populations include bone marrow mesenchymal stromal cells (BMSC), osteoblasts and adipocytes [4–6]. These cell populations are all thought to influence PCa metastasis and disease progression [7–9]. Dissecting the influence played by each stromal cell population in vivo is challenging, and this is an area where in vitro model experimentation may offer an advantage over more complex animal models. An on-going challenge is the establishment of an in vitro model that mimics the in vivo microenvironment sufficiently to yield clinically relevant results or insights. The most common tissue culture models are 2D cell monolayers grown on tissue culture polystyrene. Monolayer cultures do not facilitate tissue-like cell-cell interactions [10], and cancer cells cultured in 2D monolayers tend to be hypersensitive to anti-cancer drugs [11]. This has motivated a surge in the development of 3D cancer models that are meant to better recapitulate 3D cellular organization and complex tissue microenvironments [12, 13].

Despite the potential advantages of 3D culture models, their use in PCa drug screening remains limited. Traditional 2D tissue culture plates are inexpensive, the majority of imaging systems/protocols are designed to be compatible with 2D culture plates, and a range of automated fluidic systems are compatible with 2D culture systems. These features have not yet been efficiently integrated into 3D culture systems. For example, hydrogel matrix-based 3D cultures can be costly, they commonly suffer from significant 3D tissue size heterogeneity, and harvest from the gel is necessary for many forms of analysis [14]. Our team previously introduced the Microwell-mesh as a high throughput platform suitable for 3D tissue culture [15, 16]. The Microwell-mesh uses a microwell platform to facilitate the manufacture of hundreds of uniform multicellular 3D microtissues. It differs from previous microwell platforms in that it has a nylon mesh fixed over the microwells, and this enables retention of individual microtissues within discrete microwells even during repeat full medium exchanges. The capacity to exchange medium repeatedly is especially useful in drug testing applications. Additionally, the mesh enables establishment of microtissues from one cell type, and then the addition of a second cell type at a later time point. Because specific numbers of cells can be deposited and retained in discrete microwells, this allows the assembly of co-culture microtissues having specific co-culture cellular composition. This design makes the Microwell-mesh platform ideal for use in the simultaneous manufacture, characterization and study of the drug response of hundreds of microtissues in a high throughput manner.

An additional complexity associated with designing co-culture drug assays is that it is challenging, and potentially expensive, to specifically quantify the number of cancer cells without the co-culture population confounding this measurement. For example, simple Alamar blue metabolic readouts would include both metabolic contributions from the cancer and the stromal co-culture cell population(s), making specific cancer cell responses challenging to delineate. To overcome this barrier, we mimicked McMillin and colleagues who used a luciferase reporter system to enable the indirect estimation of cancer cell numbers in complex co-cultures via bioluminescence [17]. In our studies, the PCa cells were transduced to express a luciferase reporter, allowing us to indirectly quantify PCa cell number in complex co-cultures with stromal cell populations that did not express luciferase.

Herein, PCa cell migration and proliferation in response to bone marrow stromal cell populations cultured in 2D and 3D was contrasted. We used the Microwell-mesh system to form microtissues containing both PCa and bone marrow stromal cells, and used the luciferase reporter system to enable indirect quantification of PCa growth as well as death in response to anti-cancer drugs in complex co-cultures. The response of PCa cells to Docetaxel and Abiraterone Acetate in 2D or 3D, and in the presence or absence of stromal cells was characterized.

## Methods

### PCa cell line culture

PCa cell lines used were PC3 (purchased from ATCC® Number: CRL-1435), C42B (derived and generously shared by Dr. Chung [18]), and LNCaP cells (purchased from ATCC® Number: CRL-1740). Cell lines were authenticated at the Genomic Research Centre (GRC; Brisbane, Australia) using Short Tandem Repeat (STR) analysis. STR profiles of the cell lines were compared to the ATCC STR Database to verify cell line identity; and all cell lines showed ≥80% match to the corresponding reference STR profile. C42B were mapped back to the LNCaP STR profile, as C42B were derived from LNCaP [18]. Cells were cultured in low glucose Dulbecco’s modified Eagle’s medium (DMEM-LG; Thermo Fisher) supplemented with 10% fetal bovine serum (FBS; Thermo Fisher) and 1% penicillin/streptomycin (P/S; Thermo Fisher). For some assays, FBS was replaced with 10% charcoal stripped fetal bovine serum (CSS; Thermo Fisher) to mimic androgen deprivation conditions. Cells were grown in a cell culture incubator at 37 °C and 5% CO_2_ and 2% O_2_. All cells were passaged when monolayers reached ~ 80% confluency using 0.25% Trypsin/EDTA (Thermo Fisher).

### Human BMSC isolation, culture, characterization, and differentiation

Human bone marrow aspirates were collected at the Mater Hospital (Brisbane, Australia) from two fully informed and consenting healthy male volunteer donors. In accordance with the Australian National Health and Medical Research Council’s Statement on Ethical Conduct in Research Involving Humans, ethical approval was granted through the Mater Health Services Human Research Ethics Committee and Queensland University of Technology Ethics Committee (number: 1000000938). Aspirates were collected from the iliac crest of volunteers. Mononuclear cell isolation was achieved by density gradient centrifugation, using Ficoll-Paque Plus (GE Healthcare), as previously described [19]. Bone marrow samples were diluted 1:2 with phosphate buffered saline (PBS; Thermo Fisher) containing 2 mM EDTA (Ambion). Then the diluted sample was carefully overlayed on the Ficoll-Paque plus layer and centrifuged for 30 min at 400×*g*. The mononuclear cells collected from the interface were then washed, resuspended in DMEM-LG supplemented with 10% FBS, and 1% P/S. Cells were then cultured overnight in a humidified incubator containing 5% CO_2_ with 20% O_2_ atmosphere at 37°C. Tissue culture plastic-adherent cells were enriched by removing the medium containing non-adherent cells, and fresh culture medium added to each flask. Subsequent BMSC expansion was performed in 5% CO_2_ and 2% O_2_ atmosphere at 37°C. Cells were passaged when the monolayer reached 80% confluency using 0.25% Trypsin/EDTA. All experiments were performed using BMSC between passage 2 and 5.

The isolated cells were characterized for the expression of BMSC surface antigens; CD44, CD90, CD105, CD73, CD146, CD45, CD34 and HLADR; and mesodermal trilineage differentiation capacity and confirmed to be in accordance with the standard criteria of multipotent mesenchymal stromal cells reported previously by Dominici et al. [20].

Osteogenic and adipogenic differentiation were induced by culturing 60 × 10^3^ and 40 × 10^3^ cells/cm^2^ in osteogenic or adipogenic induction medium for 14 days, respectively. Both induction media consisted of high glucose DMEM media (DMEM-HG) containing 100 μM sodium pyruvate, 1X GlutaMax, 10% FBS and 1% P/S (all from Thermo Fisher). Additionally, osteogenic medium contained 50 μM L-ascorbic acid 2-phosphate, 100 nM dexamethasone (Sigma-Aldrich) and 10 mM β-glycerol phosphate, while adipogenic medium conatained 10 μg/mL insulin, 200 μM indomethacin, 1 μM dexamethasone (Sigma-Aldrich) and 500 μM 3-isobutyl-1-methyl xanthine (all from Sigma-Aldrich). Culture medium was replaced with fresh media twice per week.

### Generation of C42B cell line expressing luciferase-GFP (Luc-GFP)

Firefly luciferase-expressing C42B cells were generated using fresh lentiviral particles produced in-house. Luciferase-GFP (Luc-GFP) insertion constructs contained Bioluminescence Imaging Vectors (BLIV, System Biosciences) with MSCV (MSCV-Luc-GFP) promoters (Additional file 1: Figure S1). Plasmid production was achieved by using Stbl3 chemically competent *E.coli* (Thermo Fisher) as per the manufacturer’s instructions. This was followed by a purification step using the NucleoBond Xtra EF plasmid purification kit (Midi EF, Macherey-Nagel) to obtain endotoxin-free plasmid DNA. Plasmid packaging was then performed using TGEN packaging plasmid mix with the transfection reagent, Lipofectamine 2000 (Thermo Fisher). The lentiviral particles were produced by 293FT cells (Thermo Fisher) following the manufacturer’s instructions. Viral particle-containing media was then placed onto cancer cells, with the addition of 8 μg/mL polybrene (Sigma-Aldrich) to enhance transduction efficiency. Positively transduced (Luc-GFP) cells were enriched using two rounds of fluorescence-activated cell sorting (FACS; MoFlo Astrios, Beckman Coulter). This yielded a stable population of C42B cells that expressed Luc-GFP driven by a MSCV promoter. We validated the stability of luciferase gene expression in monolayer and Transwell co-culture conditions using quantitative real time-polymerase chain reaction (qRT-PCR) [15] (Additional file 1: Figure S2) and appropriate PCR primer sets (Additional file 1: Table S1).

### 3D culture system design and fabrication

An in-house fabricated microwell platform was fabricated from polydimethylsiloxane (PDMS; Slygard). PDMS microwell arrays were fabricated as described previously [11, 15]. Briefly, liquid PDMS (1:10 curing agent to polymer ratio) was permitted to cure over a patterned polystyrene mold having the negative of the microwell pattern for 1 h at 80°C. A sheet of PDMS with the microwell array pattern cast into it (each microwell had dimensions of 800 *×* 800 μm square and a depth of 500 μm) was produced and peeled from the molds. Discs (1 cm^2^ or 2 cm^2^) were punched from the PDMS sheets and then glued into culture plates with silicone glue (Selleys). For drug testing experiments, Microwell-mesh inserts were made by fixing a nylon mesh with 36 μm x 36 μm pore openings (Amazon) to the top of the microwells using silicone glue. Once the glue had cured, excess mesh was trimmed from the disc inserts using scissors. Inserts were then anchored into individual wells in 24- or 48-well plates by placing a small amount of silicone glue at the bottom of the well, and the insert pressed into the well. Plates with microwell inserts were submerged in 70% ethanol for 1 h for sterilisation, followed by rinsing of each culture well 4 times with PBS (Thermo Fisher). To prevent cell adhesion to the PDMS during culture, the PDMS microwell inserts were soaked in a sterile solution of 5% Pluronic-F127 (Sigma-Aldrich) in PBS for 10 min [21], and then rinsed 3 times with PBS before cells were seeded.

### Assembly of microtissues

**Fig. 1:**
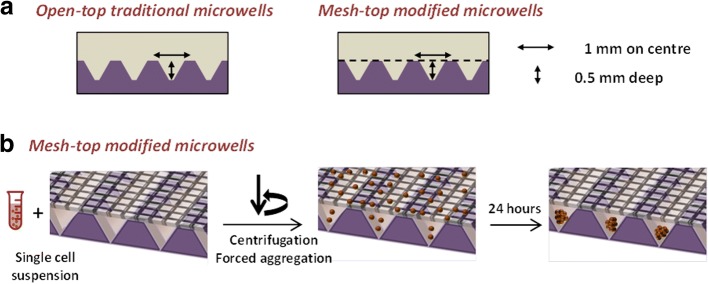
Microwell platforms and establishment of 3D microtissue culture. **a** Schematic illustration and bright field images show PDMS discs with and without the mesh, which can be inserted into 48-well tissue culture plates. **b** Schematic illustration of cell seeding in the Microwell-mesh, and microtissues retained within discrete microwells after 24 h of seeding the cells

In this study, we formed microtissues assembled from PCa, BMSC (non-induced), osteoblasts or adipocytes alone, or combination co-cultures of PCa with BMSC, osteoblasts or adipocytes. The Microwell-mesh platform was used to study PCa proliferation and drug response in direct co-cultures where multiple medium and drug exchanges were required. Fig. 1 schematically illustrates how the Microwell-mesh differs from traditional open top microwell platforms, and how centrifugation can be used to evenly distribute the seeded cell suspension into the array of microwells. Each insert had approximately 150 microwells/cm^2^, (equivalent to ~ 150 microwells per well in a 48-well plate). Seeding a different number of cells in suspension over the microwells could control the number of cells per microwell or per microtissue. Following seeding of the cell suspension, plates were centrifuged at 400 × g for 5 min to aggregate the cells uniformly at the bottom of each microwell. The aggregation of cells into microwells was visually confirmed using a microscope (Olympus CKX14), and images captured using a digital camera (Olympus DP26) and software (Olympus cellSens Entry). Plates were then transferred to a cell culture incubator maintained at 37°C and 5% CO_2_.

### PCa cell Transwell migration assay

**Fig. 2:**
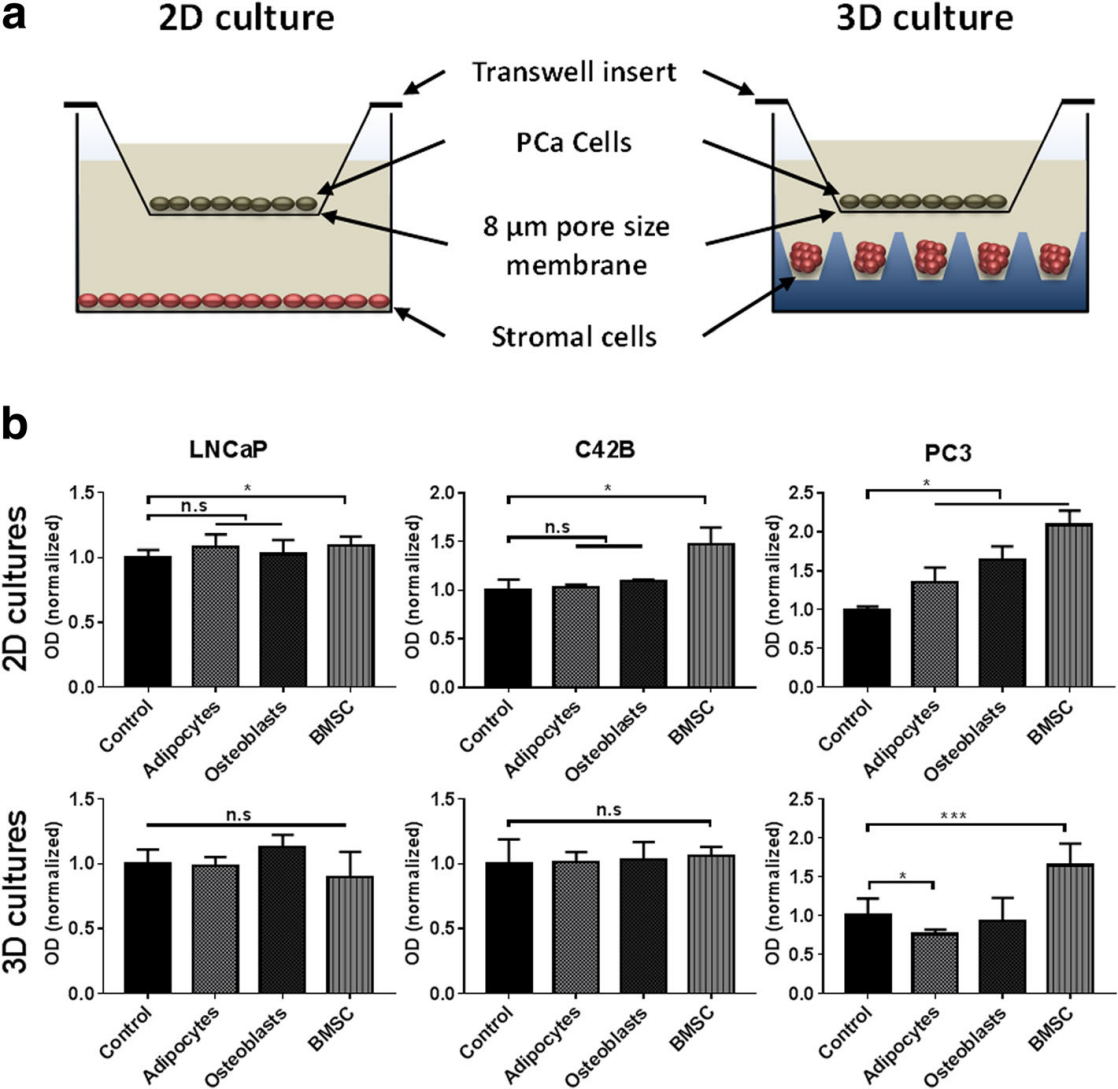
PCa migration potential in Transwell co-cultures with bone marrow stromal cells. **a** Schematic illustration of the Transwell assay. PCa cell suspensions were seeded in Transwell inserts with 8 μm pore size membrane. The co-cultures were performed over 18 h to allow PCa cell migration towards 2D monolayers or 3D microtissues of stromal cells (BMSC, osteoblasts or adipocytes). Prior to co-culture establishment, the osteoblasts and adipocytes were differentiated for 14 days using osteogenic or adipogenic induction media; and undifferentiated BMSC controls were assembled 1 day prior to initiation of the Transwell co-culture. **b** PCa cells that had migrated to the bottom surface of the Transwell membrane were stained with 0.5% crystal violet, and this was extracted and quantified. Results are represented as the mean optical densities of crystal violet extracts normalized to the control mono-cultures. Similar results were obtained in three independent experiments with two different BMSC donors, each having four replicate cultures *n* = 4. Statistical significance was performed using two-way ANOVA (* *P* < 0.05, *** *P* < 0.001 and n.s = non-significant)

We were also interested in determining if PCa cell migration towards stromal cells differed depending on the geometry of the stromal cells. In 3D microtissue co-cultures, PCa cells localized to the outside of the microtissue, but this did not provide insight into how different stromal cells might influence cell migration. To overcome this obstacle we developed a modified Transwell assay. Here, we either cultured the stromal cells as 2D monolayers or as 3D microtissues in open top microwell inserts. To quantify PCa cell migration, PCa cells were placed into Transwell inserts (pore size of 8 μm, Merck Millipore) and positioned either on top of 2D stromal cell monolayers or on top of 3D stromal cell microtissues (see Fig. 2). BMSC were seeded in 24-well tissue culture plates, and cells cultured in osteogenic or adipogenic medium for 14 days or in maintenance medium for 24 h. For 2D monolayers, 10-, 20- and 60 × 10^3^ cells/cm^2^ were seeded and cultured in the corresponding culture media. For 3D microtissues, 600 cells/microtissue were seeded in the microwell inserts anchored in the 24-well tissue culture plate as described above. Transwell inserts were seeded with 36 × 10^3^ PC3, C42B or LNCaP cells and permitted to incubate for 24 h. Inserts were then placed on top of either the 2D or 3D stromal cell populations and incubated for 18 h. At the end of the incubation period, Transwell inserts containing PCa cells were washed and moved to a new tissue culture plate. Adherent cells attached to the top surface of the Transwell insert were removed using cotton buds, while cells that had migrated to the bottom surfaces of the Transwell inserts were fixed using ice cold methanol for 15 min. Fixed Transwell inserts were immersed in crystal violet stain (0.5%, diluted in H_2_O) for 15 min. Transwell inserts were washed in running tap water to remove excess stain. Crystal violet stain was extracted from cells into 500 μl of 10% acetic acid for 10 min. The optical density (OD) of the extract was measured at 595 nm (Multiskan Go microplate spectrophotometer, Thermo Fisher). Optical density of extracts from cell-free Transwell inserts functioned as controls for empty wells. For each cell line, parallel Transwell inserts containing PCa cells not exposed to the stromal co-culture conditions functioned as baseline migration controls.

### Confocal imaging of 3D PCa-BMSC co-culture

PCa-BMSC microtissues were established using the microwell culture system. Single cell suspensions of C42B and BMSC were stained with green molecular probe (CellTrace green CFSE) and red molecular probe (CellTracker Red CMTPX; both from Thermo Fisher), respectively. A cell suspension combining the two cell types in a 1:1 ratio was generated, and seeded into 48-well tissue culture plates with microwell inserts to obtain microtissues each containing 600 cells (300 C42B cells and 300 BMSC). Following 24 h incubation at 37°C, 5% CO_2_, microtissues were collected and imaged using a Zeiss 510 Meta confocal microscope to characterize 3D cellular organization.

### Bioluminescence assay

In vitro bioluminescence of Luciferase-tagged PCa cells was used as an indirect method to estimate viable cancer cells in mono- and co-cultures. For the luciferase activity assay, D-luciferin (Promega) was added to the culture medium at a final concentration of 15 μg/mL, then incubated at 37 °C for 15 min and the bioluminescence acquired using a PHERAstar FS plate reader (BMG LABTECH).

### Cell proliferation and drug testing in direct co-culture system

PCa cell proliferation and responses to anti-cancer drugs were tested in both 2D and 3D co-cultures. In 48-well tissue culture plates, co-cultures were established in 2D monolayers or as 3D microtissue cultures. Two weeks prior to establishing co-cultures, BMSC were assembled into 3D microtissues of 300 cells/microtissue or as 2D monolayers of 60 × 10^3^ and 100 × 10^3^ cells/cm^2^ to permit differentiation to osteogenic and adipogenic lineages, respectively. At day zero, C42B Luc-GFP cells were added as a single cell suspension on top of stromal cell (BMSC, osteoblasts or adipocytes) monolayers or stromal cell microtissues in the Microwell-mesh. C42B cells were seeded at either 10 × 10^3^ cells/cm^2^ on top of stromal cell monolayers, or 300 cells per microtissue on top of established stromal microtissues.

For cell proliferation experiments, PCa cells were permitted to grow for 24 and 48 h in 2D and 3D co-cultures as mono- or co-cultures then the bioluminescence was measured as described above. Data is presented as relative bioluminescence (RLU) relative to luciferase-tagged PCa cells in mono-cultures.

Docetaxel and Abiraterone Acetate were used in the drug testing studies. Docetaxel and Abiraterone Acetate were dissolved in dimethyl sulfoxide (DMSO; all from Sigma-Aldrich), and then aliquoted and stored at − 80°C. On the day of treatment, an aliquot was thawed and diluted in culture medium to the specified concentrations.

PCa cells were permitted to adhere or aggregate into spheriods for 24 h in co-cultures. For Docetaxel treatments, all cultures were treated with the indicated concentrations starting one day after the initiation of the co-cultures, with drug treatment exposure being continuous for the next 48 h. For Abiraterone Acetate treatments, all cultures were first depleted of androgens for 48 h by replacing the FBS-supplemented culture medium with CSS-supplemented medium. Cultures were then exposed to the specified concentrations of Abiraterone Acetate for 48 h.

At the end of the drug treatment period, epifluorescence and phase contrast microscopy images were captured and bioluminescence signals from each culture were measured, as described above. Bioluminescence data is presented as a percentage of the relative bioluminescence units (RLU) compared to vehicle-treated cultures.

### Statistical analysis

Results represent two independent experiments using two BMSC donors. Each of the replicate experiments included four biological replicate cultures (*n* = 4), unless otherwise indicated. Error bars represent one standard deviation. Statistical significance of data was evaluated using two-way analysis of variance (ANOVA), using Prism software, Version 6.0 (GraphPad). *P*-values for each comparison are represented by asterisks as indicated in figure captions.

## Results

### Indirect Transwell co-culture of PCa cells with 2D and 3D bone marrow stromal cells

Using the Transwell assay, the migration of PCa cells towards BMSC, osteoblasts and adipocytes cultured in 2D monolayers or 3D microtissues was assessed following 18 h of co-culture (Fig. 2a). LNCaP, C42B and PC3 cells were used to represent or model different stages of PCa disease aggressiveness.

Of the 2D cultures, BMSC monolayers induced the greatest migration rates in all PCa cell lines tested. By contrast, the influence of osteoblasts and adipocytes on PCa migration was PCa cell line dependent. For the less aggressive cell lines, C42B and LNCaP, both osteoblasts and adipocytes had minimal influence on PCa cell migration rates. The highly aggressive bone metastatic PC3 cells demonstrated a significantly elevated migration rate towards osteoblasts and adipocytes cultured in 2D monolayers (Fig. 2b).

Unlike 2D BMSC cultures, which increased the migration of all PCa cells tested, 3D BMSC microtissues only increased the migration of PC3 cells. Indirect co-culture with 3D adipocyte microtissues decreased PC3 cell migration, and had no measurable effect on C42B or LNCaP cell migration rates. Similarly, 3D osteoblast microtissues did not increase the migration rate of any of the PCa cell lines tested (Fig. 2b).

### Spatial organization of C42B cells and stromal cells in 3D co-cultures

**Fig. 3:**
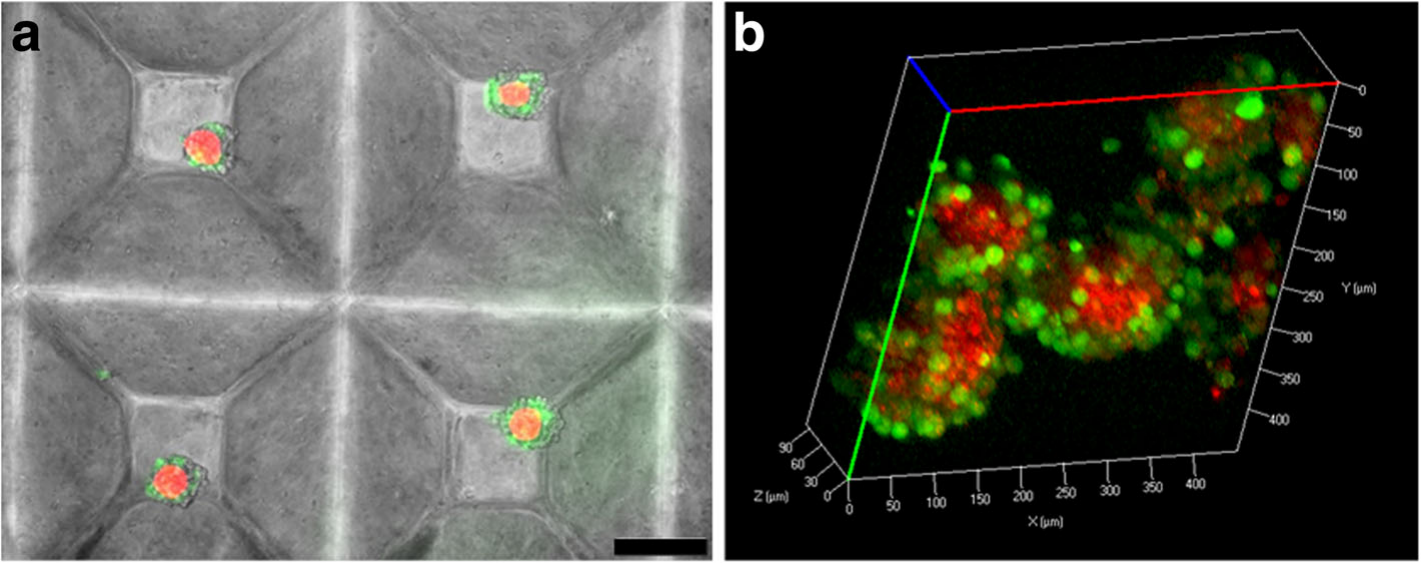
Co-culture microtissues of BMSC and C42B cells. Undifferentiated BMSC (red) and C42B cells (green) were co-cultured in the 3D microwell platform for 24 h and imaged using epifluorescence microscopy (scale bar = 200 μm) (**a**) and confocal microscopy (**b**). BMSC consistently localized to microtissue cores, while C42B cells consistently formed a shell around the BMSC cores

To characterize the spatial organization of 3D co-culture microtissues, we first labeled each cell type with differently colored fluorescent probes to enable the two cell types to be distinguished from each other. C42B cells were labeled with a green probe, while BMSC were labeled with a red probe. Figure 3a shows microtissue co-cultures formed from C42B and BMSC. Confocal images of 3D co-culture microtissues demonstrated a consistent and structured organization of the two cell types across the diameter of the microtissues. BMSC consistently localized within the core of the microtissue, whereas C42B cells were localized in the outer layer of the microtissue after 24 h of co-culture (Fig. 3b).

### C42B cell proliferation in co-cultures with bone marrow stromal cells

Conventional methods of quantifying cell proliferation, such as metabolic activity or cell viability assays, do not allow for quantification of the cell growth of a single cell population within a mixed cell population co-culture. To study C42B cell proliferation in co-cultures, we labeled the PCa cell population with a luciferase reporter system. Relative bioluminescence signal from the PCa cell populations functioned to provide an indirect estimate of the number of viable PCa cells in the different co-cultures.

To study PCa cell proliferation, we used C42B cells stably expressing Luc-GFP. These luciferase-expressing C42B cells were cultured in 2D monolayers or 3D microtissue cultures for 24 or 48 h, as either mono-cultures of PCa cells (control) or co-cultures of PCa cells with stromal cells (BMSC, osteoblasts or adipocytes). The bioluminescence assay was then performed to estimate the number of C42B cells in each culture condition at each time point.

**Fig. 4:**
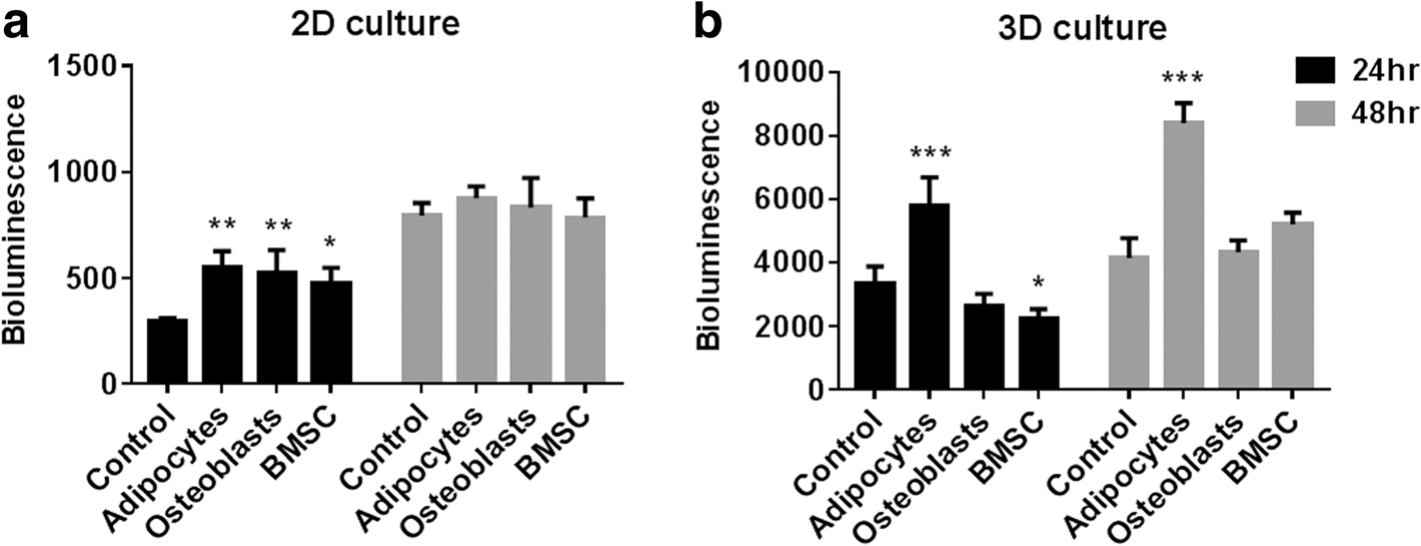
C42B cell proliferation in mono- and co-cultures with bone marrow stromal cells. Luciferase-expressing C42B cells were seeded in mono- or co-cultures with stromal cells (BMSC, osteoblasts or adipocytes) either in 2D monolayer cultures (**a**) or in 3D microtissue cultures (**b**). Results are represented as the mean bioluminescence values. Similar results were obtained in three independent experiments with two BMSC donors, each having four replicate cultures *n* = 4. Statistical significance was performed using two-way ANOVA compared to the corresponding control mono-culture value (* *P* < 0.05, ** *P* < 0.001 and *** *P* < 0.0001)

In 2D cultures, the bioluminescence values indicated a significant increase in C42B cell number in all co-culture conditions after 24 h, relative to mono-culture controls. After 48 h of culture, the effect of stromal cells on C42B cell proliferation was less pronounced. However, the overall bioluminescence after 48 h was significantly greater than after 24 h for all cultures (Fig. 4a), indicating continual cell proliferation in all culture conditions.

In 3D cultures, co-culture with adipocytes enhanced C42B cell proliferation after 24 and 48 h of culture, while co-culture with osteoblasts did not influence C42B cell proliferation rate. Despite the slight decrease in bioluminescence of C42B cells co-cultured with BMSC at 24 h, the bioluminescence tended to increase (non-significant increase) after 48 h of co-culture (Fig. 4b). Similar to 2D cultures, an overall increase in the bioluminescence of C42B cells in 3D cultures was observed at 48 h, relative to 24 h-cultures.

### C42B cell drug response in co-cultures with bone marrow stromal cells

Next, we evaluated the response of PCa cells in 2D monolayers and 3D microtissues to Docetaxel and Abiraterone Acetate, two drugs used to treat advanced PCa. Luciferase-tagged C42B cells were used in these experiments, and the bioluminescence measurements provided an indirect estimate of the viable cell number in the cultures after 48 h of drug treatments. Three replicate experiments were also performed using 2D and 3D co-culture of osteoblasts and Luciferase-expressing C42B cells. Over the total co-culture period, C42B cell viability fell dramatically, even in control co-cultures with no drug. These data suggest that long-term stability of 2D and 3D co-culture is stromal cell type dependent. In the subsequent analysis below, we focused on results derived from 2D and 3D cultures of C42B cells alone, or in co-cultured with BMSC or adipocytes.

**Fig. 5:**
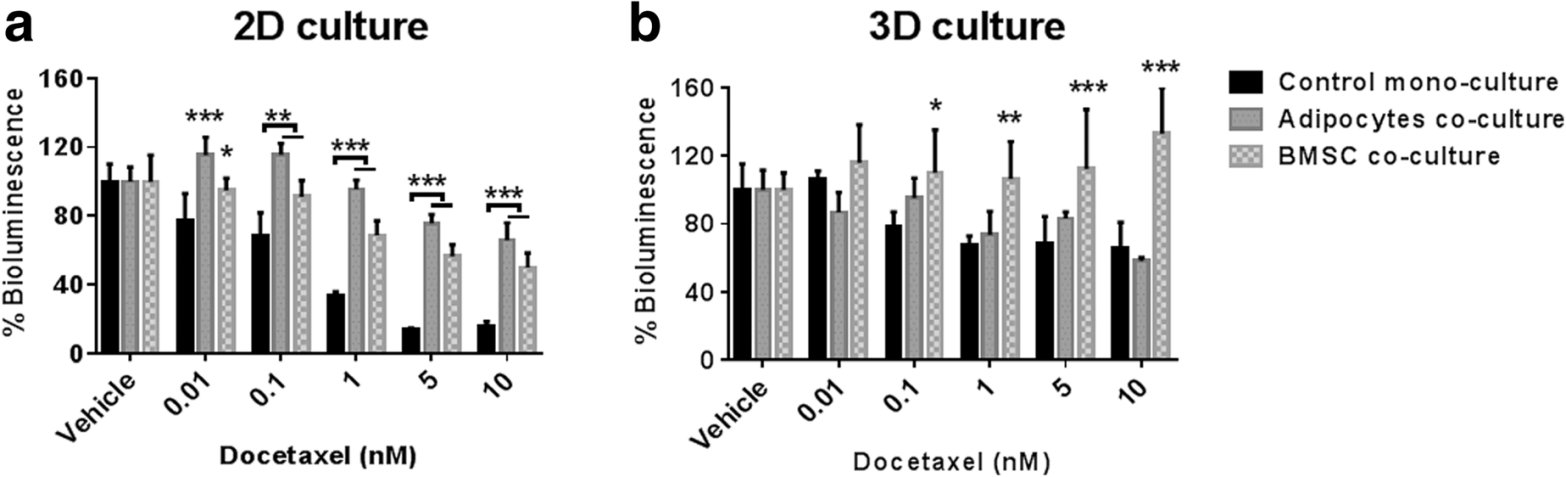
C42B cell Docetaxel drug response in mono- and co-cultures with bone marrow stromal cells. Luciferase-expressing C42B cells were seeded in mono- or co-cultures with stromal cells (BMSC or adipocytes) either in 2D monolayer cultures (**a**) or in 3D microtissue cultures (**b**). Results are represented as a percentage of the vehicle control values. Similar results were obtained in three, including with two BMSC donors, independent experiment each having four replicate cultures *n* = 4. Statistical significance was performed using two-way ANOVA compared to the corresponding control mono-culture value (* *P* < 0.05, ** *P* < 0.01 and *** *P* < 0.0001)

After 24 h of establishing the mono- and co-cultures, Docetaxel treatment was performed for 48 h. In 2D cultures, there was significantly greater bioluminescence signal from the C42B cells co-cultured with BMSC or adipocytes in all Docetaxel concentrations (0.01–10 nM) relative to the bioluminescence in mono-cultures at the same drug concentration (Fig. 5a). Unexpectedly, 3D BMSC co-cultures showed a significant increase in bioluminescence. By contrast, adipocyte co-cultures behaved similarly to corresponding mono-cultures (Fig. 5b). In general, 3D cultures demonstrated reduced sensitivity to Docetaxel in both mono- and co-cultures with BMSC or adipocytes (Fig. 5).

**Fig. 6:**
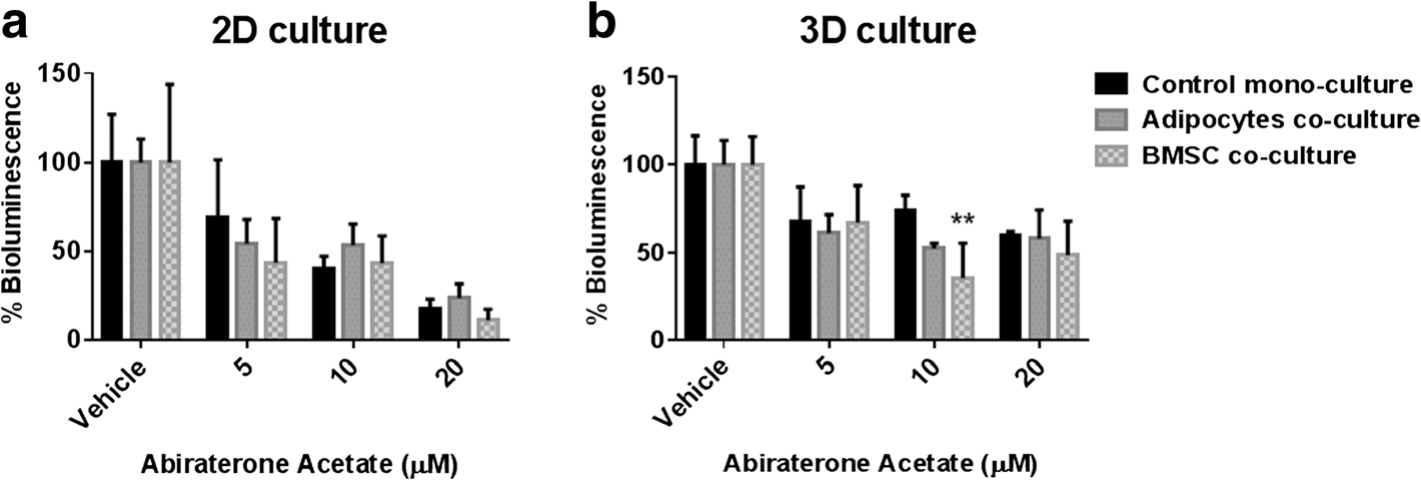
C42B cell Abiraterone Acetate drug response in mono- and co-cultures with bone marrow stromal cells. Luciferase-expressing C42B cells were seeded in mono- or co-cultures with stromal cells (BMSC or adipocytes) either in 2D monolayer cultures (**a**) or in 3D microtissue cultures (**b**). Results are represented as a percentage of the vehicle control values. Similar results were obtained in three independent experiments, including with two different BMSC donors, with each experiment having four replicate cultures *n* = 4. Statistical significance was performed using two-way ANOVA compared to the corresponding control mono-culture value (** *P* < 0.01)

For anti-androgen treatment, C42B cells were cultured in androgen deprived setting (CSS-supplemented culture media), and then treated with Abiraterone Acetate. Abiraterone Acetate is a first-in-class inhibitor of the CYP17A enzyme to prevent the biosynthesis of androgens intracellularly from their steroidal precursor [22]. The bioluminescence assay was used to assess PCa cell response in 2D and 3D mono- and co-cultures. Fig. 6 shows the bioluminescence measurements as a percentage of the corresponding vehicle control culture.

In 2D cultures, co-cultures with BMSC and adipocytes demonstrated no significant change in the anti-androgen treatment response compared to the mono-cultures of C42B cells (Fig. 6a). Similarly, 3D co-cultures did not result in change in the bioluminescence, except with BMSC co-cultures treated with 10 μM Abiraterone Acetate, which resulted in a decrease in bioluminescence (Fig. 6b). Generally, 3D mono- and co-cultures were less sensitive to increasing concentrations of Abiraterone Acetate, relative to their corresponding 2D monolayer controls.

## Discussion

Metastatic, and particularly castrate-resistant prostate cancer (CRPC), remain challenging to treat [23]. It is thought that the bone marrow microenvironment plays a pivotal role in promoting bone metastasis, possibly facilitating the transition to CRPC forms, and impacting on PCa cell drug response [24–27]. A barrier to understanding these interactions, in both drug development and testing, is the lack of in vitro models that adequately mimic aspects of the bone marrow microenvironment in a practical and high throughput manner.

Our team previously described the development of a high throughput 3D culture platform we termed the *Microwell-mesh* [15]. This platform uses a microwell insert to facilitate the manufacture of hundreds of uniform 3D multicellular microtissues. It differs from previous microwell platforms in that it has a nylon mesh fixed over the microwells, and this enables retention of individual microtissues within discrete microwells even during repeat full medium exchanges. This design is unique, and especially well suited to the assembly of 3D cultures which mimic aspects of the bone marrow microenvironment, and offers the opportunity to perform complex cultures that involve the differentiation of BMSC into different bone-like tissues, subsequent seeding of cultures with PCa cells, and the multiple medium exchanges required to study the interaction of cells and different drugs in these complex cultures. Using the Microwell-mesh to perform 3D cultures, and traditional 2D culture controls, we evaluated PCa cell migration and proliferation in response to bone marrow stromal cell populations, as well as PCa cell response to Docetaxel and Abiraterone Acetate. The goal of this study was to better understand the difference 2D and 3D stromal cell populations might have on PCa culture outcomes, and to describe models that could advance the field’s capacity to study these differences.

To study the impact of bone marrow stromal cells on the migration potential of PCa cells, we used a modified Transwell assay to quantify the migration of three different PCa cell lines towards different populations of bone marrow stromal cells (see Fig. 2). PCa cell migration rates varied depending on the aggressiveness of the PCa cell lines tested. In cell lines derived from less aggressive disease (LNCaP), relative to aggressive disease (C42B and PC3), there was a corresponding reduction in the rate of cell migration towards the bone marrow stromal cells cultured in 2D monolayers. PC3 cells, which model aggressive disease, demonstrated increased migration rates towards 2D monolayers of undifferentiated BMSC, osteoblasts and adipocytes. By contrast, PC3 cells demonstrated an increased rate of migration towards 3D osteoblasts and a reduced rate of migration towards undifferentiated BMSC or adipocytes, relative to controls. This data highlights the difference in PCa cell response depending on the PCa cell phenotype, the bone marrow stromal cell phenotype, and depending on the 2D or 3D organization of the bone marrow stromal cells. Appreciating that these factors influence outcome is an important first step that can inform our understanding and future experimental design. However, it is equally imporant to appreciate that outcomes can be influenced by the selected assay, and that not all in vitro and in vivo assays will necessarily yield the same outcome. Transwell cultures enable quantification of the influence secreted factors have on PCa cell migration, but do not necessarily provide insight into how stromal cell-specific matrix or bound factors may directly influence PCa cell behavior. Thus, Transwell assay outcomes provide only part of the necessary insight.

Next, we investigated how 2D or 3D culture of different bone marrow stromal cell populations impacted on C42B cell proliferation. C42B cell proliferation was greater when these cells were seeded on 2D monolayers of undifferentiated BMSC, adipocytes or osteoblasts (see Fig. 4a). This result is consistent with the general view that stromal cells can play a supportive role in co-cultures, and especially those that mimic aspects of the support environment found in the bone marrow niche [28, 29]. This result is also consistent with a previous report indicating that BMSC-conditioned media supports PCa cell proliferation [30]. In 3D co-cultures, only adipocytes were found to drive significant increases in C42B cell proliferation (see Fig. 4b). This substantial difference in 2D and 3D culture outcomes is interesting, as it indicates that geometry can significantly impact co-culture outcomes. Future studies might compare the secretion profiles of BMSC, adipocytes or osteoblasts in 2D and 3D, with the hypothesis that culture geometry significantly influences what factors are produced by the stromal cell populations. There are already a number of studies that suggest the secretome of BMSC is more supportive when BMSC are assembled into spheroids [31, 32]. Characterizing precise changes in the gene expression or secretion profile of the mesenchymal and PCa cells assembled into spheroids would require digesting the co-culture microtissues into single cell suspensions, followed by cell sorting and then gene or protein analysis. The considerable processing steps and time would likely confound the results. Thus, within this manuscript we focused on platform development and phenomenological characterization of PCa growth and drug response as influenced by the presence or absence of different mesenchymal cell populations. Equally valuable, would be to compare how 2D and 3D co-cultures influence the proliferation of primary PCa cells. Primary PCa cells are particularly challenging to culture in vitro [33], but their response to co-culture might be more meaningful than the response from an adapted cell line. We see these important investigations as beyond the scope of this manuscript, but obvious opportunities that could be explored using the Microwell-mesh as a tool to facilitate these important next steps.

In our studies, we found that the drug response of C42B cells differed in 2D and 3D co-cultures, and that response varied depending on the stromal cell population used in the co-culture (see Figs. 5 and 6). Collectively, the presence of BMSC or adipocytes in 2D or 3D co-culture reduced C42B cell sensitivity to Docetaxel, a drug commonly used to treat metastatic disease. Other groups have reported similar observations [17, 34, 35], suggesting that bone marrow stromal cells likely do influence PCa cell drug sensitivity. In contrast to tests conducted with Docetaxel, the drug response of C42B cells to Abiraterone Acetate did not appear to be influenced by the presence or absence of bone marrow stromal cells. However, the organization of C42B cells into 3D cultures did reduce these cells sensitivity to Abiraterone Acetate, relative to 2D cultures. This outcome suggests that relative proliferation rates, which are generally reduced in 3D cultures [36, 37], may play a greater role than the presence or absence of stromal cells in influencing the impact of anti-androgen treatment.

## Conclusions

Overall, our results indicate that C42B cell behaviour can vary depending on the phenotype and geometry of bone marrow stromal cells included in co-culture. Through this work we have described the development of a 3D co-culture platform, the Microwell-mesh, that enables the assembly of 3D bone stromal cell microtissues, the subsequent introduction of PCa cells, and then evaluation of PCa cell proliferation or drug response. Using this novel 3D culture platform, we show that PCa cell response to drugs varies considerably in 2D and in 3D, and culture outcomes are also stromal cell-type dependent. This 3D culture tool provides more complex in vitro analysis, and will hopefully lead to the more efficient identification of improved PCa treatment strategies.

## Additional file

Additional file 1: **Figure S1.** Restriction map of MSCV-Luc-GFP plasmid. **Figure S2.** Luciferase gene expression in C42B-MSCV cell lines. **Table S1.** Primers and annealing temperatures used for qRT-PCR. (PDF 609 kb)

## Abbreviations

2D: Two-dimensional
3D: Three-dimensional
BMSC: Bone marrow mesenchymal stromal cells
CRPC: Castrate-resistant prostate cancer
CS-BLI: Cell-specific bioluminescence assay
CSS: Charcoal-stripped serum
HSC: Haematopoietic stem cell
Luc-GFP: Luciferase-GFP
PCa: Prostate cancer
