# Supplementary material for: Using high throughput microtissue culture to study the difference in prostate cancer cell behavior and drug response in 2D and 3D co-cultures
Source: BMC Cancer. 2018 May 24;18:592. doi: 10.1186/s12885-018-4473-8 (PMC5968610; doi:10.1186/s12885-018-4473-8)
Supplement: Supplementary file 1 — Figure S1. Restriction map of MSCV-Luc-GFP plasmid. Figure S2. Luciferase gene expression in C42B-MSCV cell lines. Table S1. Primers and annealing temperatures used for qRT-PCR. (PDF 609 kb) [file 12885_2018_4473_MOESM1_ESM.pdf]

Additional file 1

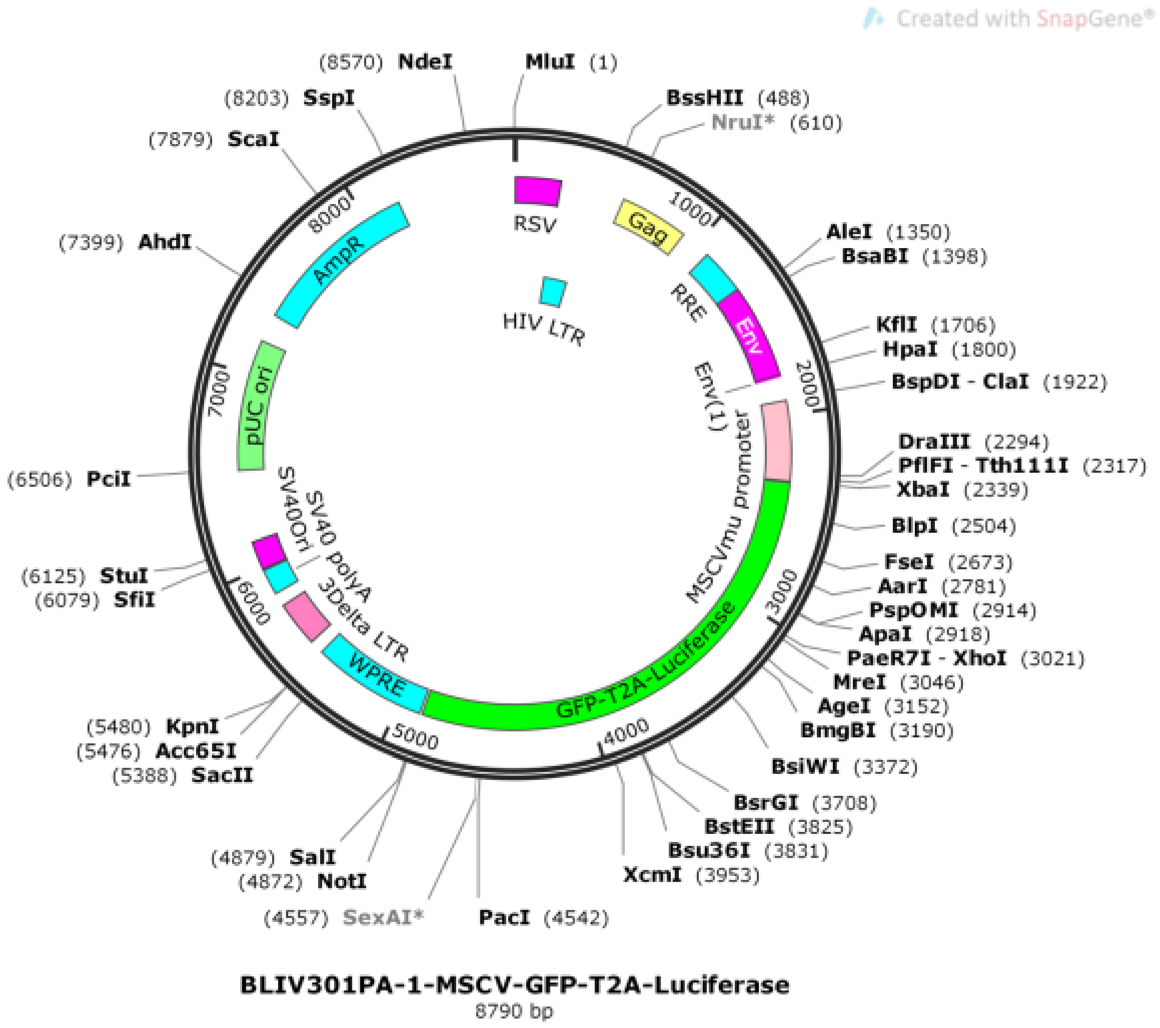

Figure S1. Restriction map of the plasmid used in the transduction and manufacture of LUC-GFP expressing C42B PCa cells. Plasmid was designed by System Biosciences (Bioluminescence Imaging Vectors, BLIV).
